# Comparison of Crimean-Congo Hemorrhagic Fever Virus and Aigai Virus in Life Cycle Modeling Systems Reveals a Difference in L Protein Activity

**DOI:** 10.1128/jvi.00599-22

**Published:** 2022-06-13

**Authors:** Matthew J. Pickin, Stéphanie Devignot, Friedemann Weber, Martin H. Groschup

**Affiliations:** a Institute of Novel and Emerging Infectious Diseases, Friedrich-Loeffler-Institut, Greifswald-Insel Riems, Germany; b Institute for Virology, FB10-Veterinary Medicine, Justus-Liebig University, Giessen, Germany; University of Kentucky College of Medicine

**Keywords:** CCHFV, AP92, Europe II, orthonairovirus, Aigai virus, genotype VI, *Nairoviridae*, Crimean-Congo

## Abstract

Crimean-Congo hemorrhagic fever virus (CCHFV) is a tick-borne orthonairovirus that causes a severe, often fatal, hemorrhagic disease throughout Africa, Asia, and Southeast Europe. A wide variety of strains are circulating in the field which broadly correlate to their geographic distribution. The viral determinants of pathogenicity remain unclear, as does the contribution of strain-specific differences to pathology. Aigai virus (AIGV) is a closely related virus (formally designated CCHFV genotype VI, Europe II, or AP92-like virus), which has been proposed to be less virulent than CCHFV. However, the molecular details leading to potential differences in virulence are unknown. To explore if differences exist, life cycle modeling systems, including both a minigenome and a transcriptionally competent virus-like particle assay, were developed for AIGV to allow the comparison with the CCHFV reference IbAr10200 strain. Using this approach, we could demonstrate that AIGV exhibits lower viral gene expression than the reference strain of CCHFV. Subsequent systematic exchange of viral components revealed that the L protein is responsible for the observed differences in gene expression and that the interferon (IFN) antagonistic activity of the ovarian tumor-type protease domain is not responsible for this effect.

**IMPORTANCE** Crimean-Congo hemorrhagic fever virus (CCHFV) is the cause of severe hemorrhagic disease, which is often fatal. Present throughout Africa, Asia, and Southeast Europe, a diverse number of viral genotypes exist. However, the viral determinants of pathogenicity remain unclear. It has been proposed that the closely related Aigai virus (AIGV) may be a less virulent virus. Here, using newly developed and improved life cycle modeling systems we have examined potential differences between the CCHFV reference strain, IbAr10200, and AIGV. Using this approach, we identified lower viral gene expression driven by the AIGV viral polymerase as a major difference which may be indicative of lower virulence.

## INTRODUCTION

Crimean-Congo hemorrhagic fever virus (CCHFV) (order *Bunyavirales*, family *Nairoviridae*, genus *Orthonairovirus*) is a causative agent of Crimean-Congo hemorrhagic fever, a severe and often fatal disease. Widespread throughout Africa, Asia, and Southeast Europe, CCHFV is found almost everywhere ticks of the *Hyalomma* genus are found, which represent the primary host and vector of CCHFV (1). Due to the threat it poses to public health and the absence of countermeasures, CCHFV has been listed as a priority disease on the R&D Blueprint of the World Health Organization (2).

Like all orthonairoviruses, CCHFV has a negative-sense RNA genome, which is split across three segments known as the large (L), medium (M), and small (S) segments. The S segment encodes the nucleoprotein (N), which encapsidates the genome segments to form nucleocapsids that serve as the templates for transcription and replication (3). Encoded on the M segment, the glycoproteins are produced as a glycoprotein precursor (GPC) polyprotein, which is cleaved and posttranslationally modified to give rise to the structural glycoproteins Gn and Gc, as well as a number of nonstructural proteins of unknown function (3). The L protein, which is encoded on the L segment, is the viral RNA-directed RNA polymerase (vRdRp), which mediates replication of and transcription from the genome segments (4). Noncoding regions (NCRs) flanking the open reading frame (ORF) of each genome segment direct RNA synthesis. Viral transcription is primed using short fragments of RNA which are “cap-snatched” from host mRNAs, a process also mediated by the L protein (5).

The L protein of CCHFV is unusually large compared with polymerases of other viruses of the *Bunyavirales* order: it is, for example, almost double the length of the phlebovirus L proteins (6). In alignment with the other L proteins of the *Bunyavirales*, significant expansions exist between all the conserved regions (6). The most studied of these expansions is an N-terminal extension, which contains an ovarian tumor-type protease (OTU) domain that cleaves ubiquitin and ISG15, which functions in counteracting the innate immune response (7) and regulating polymerase activity (8, 9). In addition to the activity of the OTU domain and traditional vRdRp roles, it is believed that the uncharacterized regions of the L protein may have as yet undescribed additional functions.

CCHFV is reported to have the highest genetic diversity of the known arboviruses (1). Based on the available sequences, CCHFV viruses have been divided into distinct genotypes. Depending on the segment used to classify the genotype, there are five (M or L segment) or six (S segment) genotypes. As they typically align with their geographical distribution, the genotypes are often referred to by the name of their main location (Africa I, II, and III; Asia I and II; and Europe I and II) (1, 10). The role of this diversity in pathogenicity during human infection is not fully understood. In animal models, some strain-specific differences in pathogenicity have been identified, with the reference IbAr10200 (Africa I) strain causing rapid (<4 days) universal lethality in type I interferon receptor knockout (IFNAR^−/−^) mice, while the Hoti (Europe I) strain has a more progressive disease course, although it is universally lethal by day 8 in the same model (11).

Aigai virus (AIGV), previously classified as CCHFV genotype VI, Europe II, or AP92-like CCHFV, has recently been designated a member of a new viral species within the *Orthonairovirus* genus. It has been postulated that AIGV and related viruses may be less virulent than CCHFV despite their close phylogenetic relationship (12). This proposal is based on a number of observations: AIGV (formally CCHFV Europe II) cocirculates with CCHFV Europe I viruses in the Balkans and Turkey, where relatively high CCHFV seroprevalence in both humans and livestock is seen (note that AIGV is included as “CCHFV” in these data) (13, 14). Although these regions have a high occurrence of CCHF, these cases are almost exclusively caused by CCHFV Europe I viruses. Despite this, three human CCHF cases caused by AIGV infections have been reported, one of which was fatal, demonstrating that these viruses are not apathogenic (15–17). Crucially, the areas where AIGV circulates have relatively advanced health care systems, and as such, the low incidence of CCHF cases caused by AIGV is unlikely to be due to a lack of screening. However, as the serological surveys carried out to date are unable to distinguish between CCHFV and AIGV, it is not possible to determine if the paucity of CCHF cases caused by AIGV is due to fewer infections occurring (i.e., reduced viral fitness in humans) or due to the less virulent nature of AIGV.

As CCHFV is classified as a risk group 4 agent, work with infectious virus is limited to the few sites with access to maximum containment laboratories. To facilitate research of risk group 4 agents, systems collectively known as life cycle modeling systems have been developed for many high-containment viruses, which allow investigation of viral processes at lower biosafety levels (5, 18, 19). These systems have the additional benefit of allowing the dissection of individual processes of the viral life cycle.

Like most life cycle modeling systems, the CCHFV systems hinge around a minigenome which consists of a reporter gene like the *Renilla* luciferase (rLuc) gene flanked by the NCRs of one of the genome segments. The minigenome is carried on a plasmid under the control of a T7 promoter, which upon cotransfection with T7 DNA-directed RNA polymerase (T7pol) results in the production of the minigenome. By including plasmids expressing N and L proteins of CCHFV, the minigenomes are encapsidated by N protein, which then serves as the template for RNA synthesis directed by the L protein. Both replicase and transcriptase activity occur, leading to rLuc expression, which can be measured in a luciferase assay. Under these conditions, known as the minigenome assay, luciferase activity can be used as a measure of viral replicase and transcriptase activity.

Inclusion of a plasmid expressing the CCHFV GPC in the transfections for the minigenome assay results in the packaging of the minigenome-containing ribonucleocapsids and production of transcriptionally competent virus-like particles (tcVLPs) (5). The tcVLPs can infect new cells and reinfect the transfected producer cells, which results in enhanced reporter gene activity. In the transfected producer, or passage 0 (p0), cells, reporter gene activity is the product of viral protein-directed replicase and transcriptase activity as well as viral budding and entry processes. The tcVLP-containing supernatants can also be harvested and used to infect other populations of cells. Depending on the state of these cells, various stages of the viral life cycle can be modeled. Pretransfection of cells with plasmids expressing N, GPC, and L, known as p1 cells, allows modeling of most stages of the viral life cycle as entry and uncoating are modeled in addition to budding and the polymerase activities. Passaging of tcVLP in this way can be repeated with subsequent passages (p2, p3, etc.) on similarly pretransfected cells to model serial passaging of virus. Alternatively, transfection of cells with N and L expression plasmids, prior to tcVLP infection, termed indicator cells, models viral entry and replicase activity without the influence of nonviral encapsidation of T7pol-derived minigenomes. Indicator cells can also be used as a surrogate for quantifying the number of tcVLPs produced in the producer/p0 cells.

The aim of this study is to use life cycle modeling systems to establish possible differences in the fundamental molecular biology of AIGV (AP92 strain) in comparison with CCHFV (IbAr10200 strain), which may support the proposal that AIGV is a low-virulence virus.

## RESULTS

### Optimization of the CCHFV minigenome assay.

**FIG 1:**
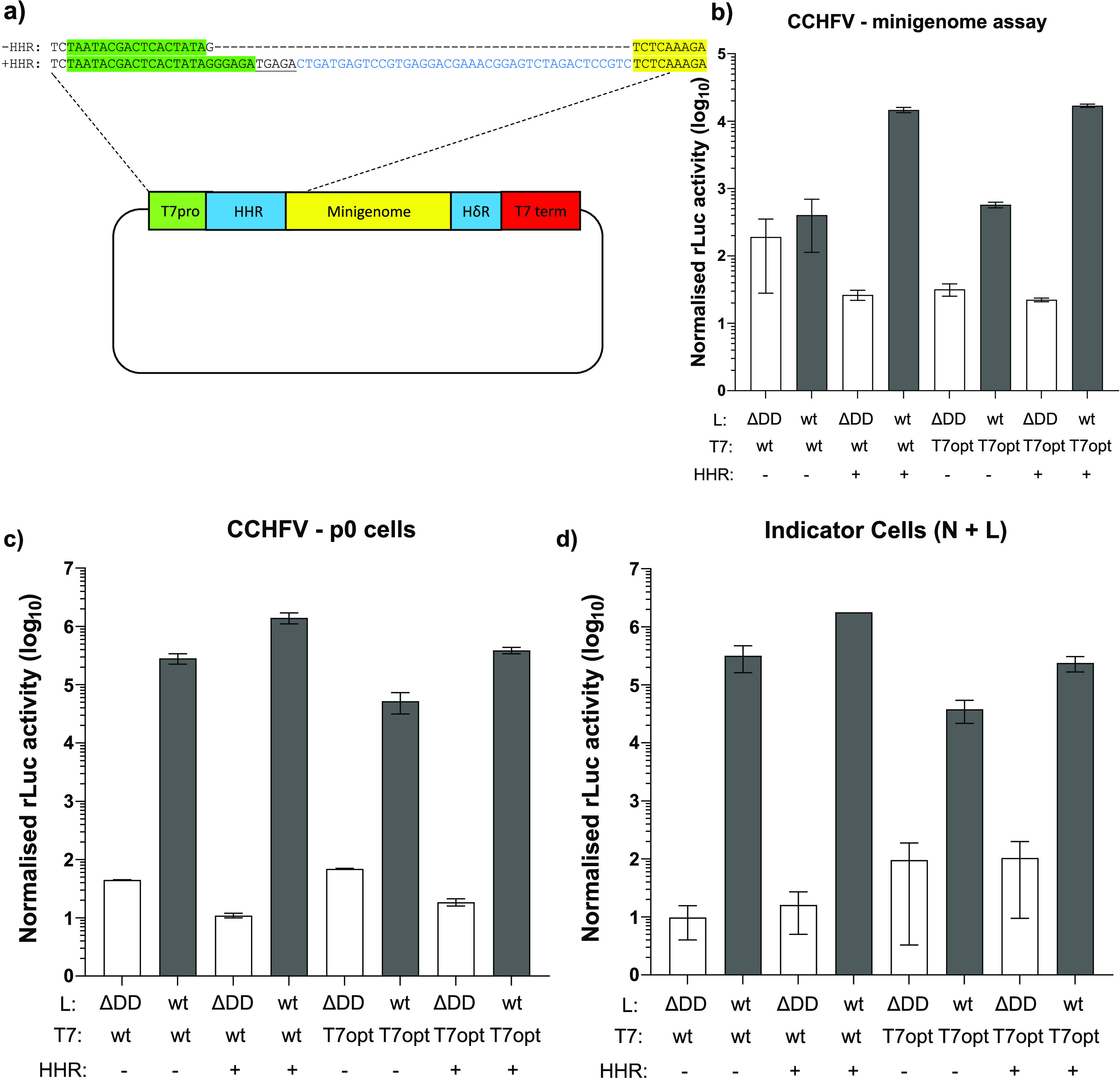
Optimizing the CCHFV minigenome assay in Huh7 cells. (a) Schematic of minigenome plasmid used in this comparison. T7 promoter (green) and terminator (red), hammerhead and hepatitis delta 1 virus ribozymes (blue), and minigenome sequence (yellow) are highlighted. (b) Huh7 cells were transfected with plasmids containing a CCHFV L *Renilla* luciferase minigenome either with (+) or without (−) a hammerhead ribozyme, wild-type (wt) or codon-optimized (T7opt) T7 RNA polymerase, wild-type L protein (wt; gray bars) or a polymerase-deficient mutant (ΔDD; white bars), N protein, and a constitutively expressed firefly luciferase. Dual-luciferase assays were carried out 48 h posttransfection, and the *Renilla* luciferase activity was normalized to firefly luciferase activity. (c) Transfections were carried out as described for panel b; however, a plasmid expressing CCHFV GPC was included under all conditions. Seventy-two hours posttransfection, tcVLP-containing supernatants were harvested, and a dual-luciferase assay was carried out with *Renilla* luciferase activity normalized to the firefly luciferase activity. (d) tcVLP-containing supernatants harvested from the cells described for panel c were used to “infect” a fresh set of Huh7 cells, which had 24 h previously been pretransfected with plasmids expressing CCHFV L proteins, CCHFV N protein, and a constitutively expressed firefly luciferase. Twenty-four hours after tcVLP infection, a dual-luciferase assay was carried out with *Renilla* luciferase activity normalized to the firefly luciferase activity. All data presented are representative data from experiments carried out in duplicate, with error bars showing standard deviation.

To allow a meaningful comparison of the CCHFV and AIGV in the life cycle modeling systems, we first established minigenome assays in Huh7 (human hepatoma) cells, the cell line required for successful propagation of CCHFV-derived tcVLPs (5). It has previously been reported that CCHFV minigenome assays in Huh7 cells resulted in insufficient luciferase activity to be a useful assay (5). These results could be confirmed, as transfecting Huh7 cells with the plasmids required for a minigenome assay resulted in only an ~15-fold increase in luciferase values over the negative control (Fig. 1b). Hoping to improve these values, we modified the existing system in two ways. The first modification was the use of a codon-optimized T7 RNA polymerase (T7opt)-expressing plasmid, which resulted in a small (~0.5-fold) increase in luciferase activity. The second modification was to the minigenome-expressing plasmid, whereby the minimal T7 RNA promoter was exchanged with a full-length T7 RNA promoter with a hammerhead ribozyme (HHR) inserted downstream between the end of the promoter and the start of the minigenome (Fig. 1a). Using the new minigenome plasmid, a large increase in luciferase values was seen, with ~1,000-fold higher values than the negative control. In combination, the two modifications resulted in a large increase in reporter activity over the previous conditions; however, the increase was smaller than using the HHR-incorporating minigenome in combination with the wild-type T7-expressing plasmid (Fig. 1b).

Next, we ensured that the two modifications made to enhance the minigenome assay were not detrimental to the CCHFV tcVLP assay (Fig. 1c and d). In this setting, the use of T7opt was actually detrimental, as it resulted in lower luciferase values in the assay, but at the same time increased reporter activity in the negative-control samples. This result was seen in both the donor and indicator cells, leading to a reduced dynamic range of the assay. In contrast, the introduction of the HHR-containing minigenome plasmid resulted in an ~10-fold increase in luciferase values, with a similar or lower background luciferase value in the negative-control wells in the indicator and donor cells, respectively. In this context, the use of the two modifications in combination showed little or no improvement over the original system. Based on the combined results from the two assays, it was decided to proceed using the HHR-containing minigenome plasmid in combination with the wild-type T7pol-expressing plasmid.

### Comparing CCHFV and AIGV strains in a tcVLP assay.

To establish the AIGV tcVLP and minigenome assays, the required AIGV-derived plasmids were produced after carrying out RT-PCRs on RNA extracted from cells infected with AIGV. The amplified products were ultimately inserted into the same vector used in the previously reported CCHFV-based life cycle modeling systems to allow a meaningful comparison (5). A ΔDD polymerase-deficient AIGV L protein-expressing plasmid was also produced to serve as the negative control. To produce the AIGV-derived minigenome, fragments incorporating the NCRs from the AIGV L segment were synthesized and inserted into the minigenome-containing plasmid. The synthesized fragments also included the optimal T7 promoter (T7pro) with the HHR to allow for a direct comparison with the new HHR containing the CCHFV-L-derived minigenome.

**FIG 2:**
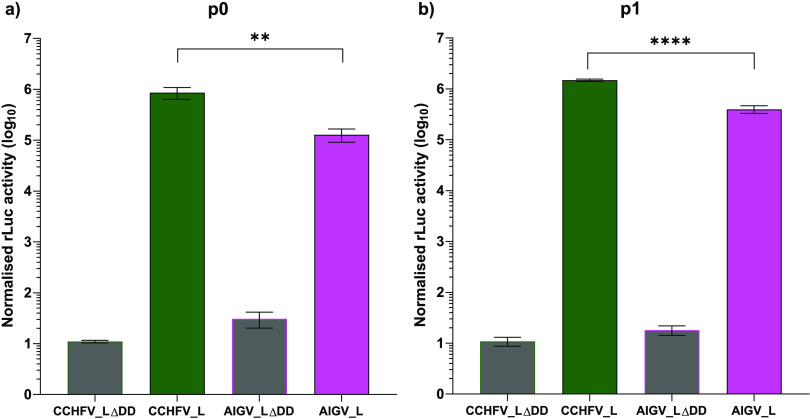
Comparison of CCHFV and AIGV in tcVLP assays. (a) Huh7 cells were transfected with a set of plasmids individually containing an L segment *Renilla* luciferase minigenome with a hammerhead ribozyme, wild-type T7 RNA polymerase, either wild-type L protein (Lwt; full color bars) or a polymerase-deficient mutant (LΔDD; gray bars), a GPC, an N protein, and a constitutively expressed firefly luciferase. The viral sequences were derived from either CCHFV (green) or AIGV (pink). Supernatants were harvested 72 h posttransfection, and dual-luciferase assays were carried out with *Renilla* luciferase activity normalized to firefly luciferase activity. (b) Huh7 cells transfected with plasmids expressing L protein, GPC, N protein derived from CCHFV (green) or AIGV (pink), and firefly luciferase 24 h previously were “infected” with tcVLP-containing supernatants harvested from the cells in panel a. CCHFV- and AIGV-derived tcVLPs were used to infect cells expressing viral protein derived from the same virus. For all data presented, *n* = 8, with error bars showing standard deviation. Statistical analysis was carried out with an unpaired *t* test. **, *P* < 0.01; ****, *P* < 0.0001.

Having produced the required plasmids, we carried out a direct comparison of the CCHFV- and AIGV-derived tcVLP systems. To do this, Huh7 cells were transfected with the required plasmids for generation of tcVLPs derived from either CCHFV or AIGV. In the transfected, tcVLP-producing (p0) cells, significantly lower (~1-log_10_) luciferase values were seen when AIGV-derived tcVLPs were produced compared to CCHFV-derived tcVLPs (Fig. 2a). The negative-control LΔDD-transfected wells, did not show such differences. Equivalent results were seen following infection of cells expressing N, GPC, and L (p1 cells) with tcVLPs (Fig. 2b). These results suggest that at least one process in the viral life cycle is impaired in AIGV relative to CCHFV.

**FIG 3:**
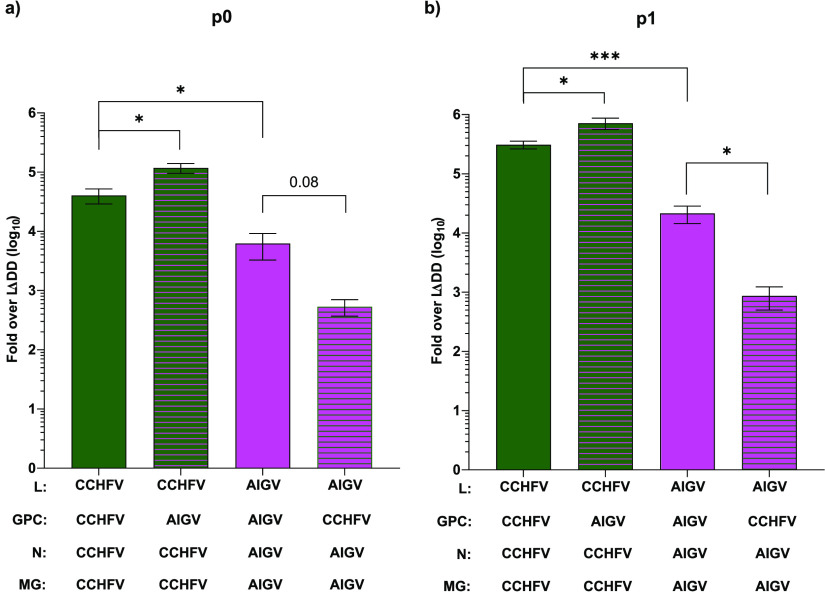
Exchanging GPC between CCHFV and AIGV in tcVLP assays. (a) Huh7 cells were transfected with plasmids required to produce tcVLPs (Fig. 2) derived from either CCHFV or AIGV as annotated below the graph. After 72 h, the supernatants were harvested, and dual-luciferase assays were carried out with the cell lysates. The data shown are the fold increase in normalized luciferase values over the polymerase-deficient mutant of the respective virus. (b) Supernatants harvested from the cells in panel a were used to “infect” Huh7 cells 24 h previously transfected with plasmids expressing L protein, GPC, N protein, and a constitutively expressed firefly luciferase. After 72 h, dual-luciferase assays were carried out on the cell lysates. For all data presented, *n* = 6, with error bars showing standard deviation. Statistical analysis was carried out with an unpaired *t* test. *, *P* < 0.05; ***, *P* < 0.001.

To explore the cause of the reduced activity seen using the AIGV-derived system, we repeated the tcVLP assay, this time including wells where the plasmids expressing the GPC were exchanged between the two strains (Fig. 3). In p0 cells, the use of AIGV GPC in an otherwise CCHFV setup resulted in a small increase in luciferase activity relative to the well receiving only CCHFV-derived components (Fig. 3a). The reciprocal case, with the use of CCHFV GPC in combination with the AIGV components, resulted in a reduction of luciferase values relative to the all-AIGV setting. Again, a significant reduction relative to CCHFV was seen in the all-AIGV tcVLP wells (Fig. 3). These results suggest the cause of reduced luciferase activity was due to a component of the viral replicative machinery, namely, the N or L protein, or alternatively an element in the NCRs. These results from the p0 cells were also replicated in the p1 cells, where a clearer difference was seen, further supporting this conclusion (Fig. 3b).

### Comparing CCHFV and AIGV in a minigenome assay.

**FIG 4:**
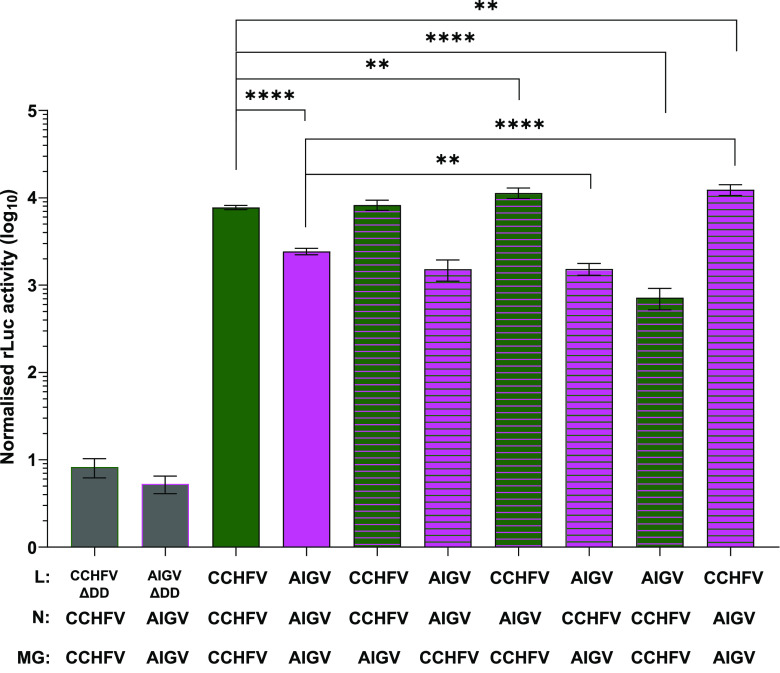
Exchanging CCHFV and AIGV components in minigenome assays. Huh7 cells were transfected with plasmids containing an L segment *Renilla* luciferase minigenome with a hammerhead ribozyme, wild-type T7 RNA polymerase, either wild-type L protein (Lwt; colored bars) or a polymerase-deficient mutant (ΔDD, gray bars), an N protein, and a constitutively expressed firefly luciferase. The viral sequences were derived from either CCHFV or AIGV as indicated below. Cells were lysed 48 h posttransfection, and dual-luciferase assays were carried out with *Renilla* luciferase activity normalized to firefly luciferase activity. For the data presented, *n* = 4, with error bars showing standard deviation. Statistical analysis was carried out with an unpaired *t* test. **, *P* < 0.01; ****, *P* < 0.0001.

To confirm our conclusions from the tcVLP assays, we carried out a comparison of the CCHFV and AIGV components in a minigenome assay. As expected, the luciferase values were significantly reduced in the AIGV minigenome assay compared with the activity seen in the CCHFV minigenome assay, with activity 3-fold higher in the CCHFV assays (Fig. 4). To further explore the cause of this difference, each of the components was systematically exchanged. The exchange of minigenomes between the two strains resulted in little change. A small reduction in activity was seen using the CCHFV minigenome in combination with the AIGV N and L proteins; however, the large difference remained between wells containing CCHFV N and L compared to those receiving AIGV N and L. The use of AIGV N alongside CCHFV L and minigenome resulted in a small increase relative to the all-CCHFV minigenome assay. The reverse was also true with CCHFV N used alongside AIGV L and minigenome, resulting in a small decrease relative to a completely AIGV minigenome assay. However, exchange of the L proteins between the CCHFV and AIGV minigenome assays resulted in a significant change. The use of AIGV L with CCHFV N and minigenome resulted in a significant drop in luciferase activity relative to the all-CCHFV minigenome assay. The reduction in activity seen using AIGV L was larger than that seen comparing the all-AIGV minigenome assay to the all CCHFV system. The converse was also true, with introduction of CCHFV L into an otherwise AIGV minigenome assay resulting in luciferase values significantly higher than those in an all-AIGV system. The values seen using CCHFV L with AIGV N and minigenome exceeded those seen in the all-CCHFV system. Taken together, these results show that a difference in an L protein activity between CCHFV and AIGV is responsible for the difference in luciferase activity in both the tcVLP and minigenome assay. The data also suggest that the other components of the AIGV basic replicative machinery enhance this activity, but not at a level that sufficiently compensates.

### Differences in protein levels do not contribute to the reduced L protein activities.

**FIG 5:**
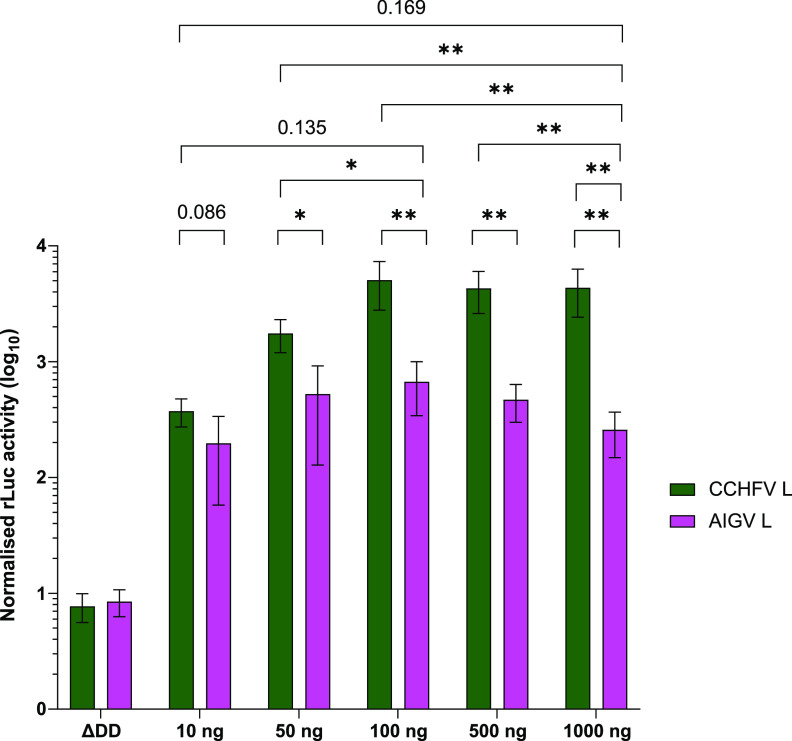
Titration of CCHFV and AIGV L in minigenome assays. Huh7 cells were transfected with plasmids containing an CCHFV L segment *Renilla* luciferase minigenome with a hammerhead ribozyme, wild-type T7 RNA polymerase, CCHFV N protein, and a constitutively expressed firefly luciferase in combination with a plasmid expressing either CCHFV L (green) or AIGV L (pink) at various amounts indicated below. The polymerase-deficient (ΔDD) negative control for the respective strains was included at 500-ng amounts, and total plasmid DNA was normalized using an empty pCAGGS vector. The cells were lysed 48 h posttransfection, and dual-luciferase assays were carried out with *Renilla* luciferase activity normalized to firefly luciferase activity. For the data presented, *n* = 4, with error bars showing standard deviation. Statistical analysis was carried out with an unpaired *t* test. *, *P* < 0.05; **, *P* < 0.01.

One possible explanation for the difference in the activity seen between the two L proteins is that the AIGV L is expressed at lower levels due to, for example, lower translational efficiency or more rapid degradation of either the mRNA or protein. To examine whether expression levels cause the observed differential activity, we carried out a titration of the L-encoding plasmids, both CCHFV and AIGV, in the context of a CCHFV minigenome assay (Fig. 5). The titration was carried out from 10 ng to 1,000 ng of L plasmid, with total plasmid amounts equalized using an empty vector. At all L plasmid amounts, lower activity was seen in wells receiving AIGV L-encoding plasmids compared with those receiving CCHFV L. These differences reached statistical significances at all amounts of L plasmids, except for 10 ng, although reduced activity was still observed. Peak luciferase activity using AIGV L was also significantly lower than that of the CCHFV L, which was seen in both cases at 100 ng of L plasmid. The use of 50 ng of CCHFV L plasmid resulted in activity higher than peak AIGV L activity, and higher activity was seen titrating CCHFV L plasmid down to 50 ng compared with 1,000 ng of AIGV L plasmid, with roughly similar values seen at 10 ng of CCHFV L plasmids. Although not directly examining protein levels, these data suggest that the difference in reporter gene activity is not due to protein expression levels or stability, but rather is due to differences in an inherent property of the L protein of the two strains.

### Differential CCHFV L protein activity is distinct from IFN antagonistic effects.

**FIG 6:**
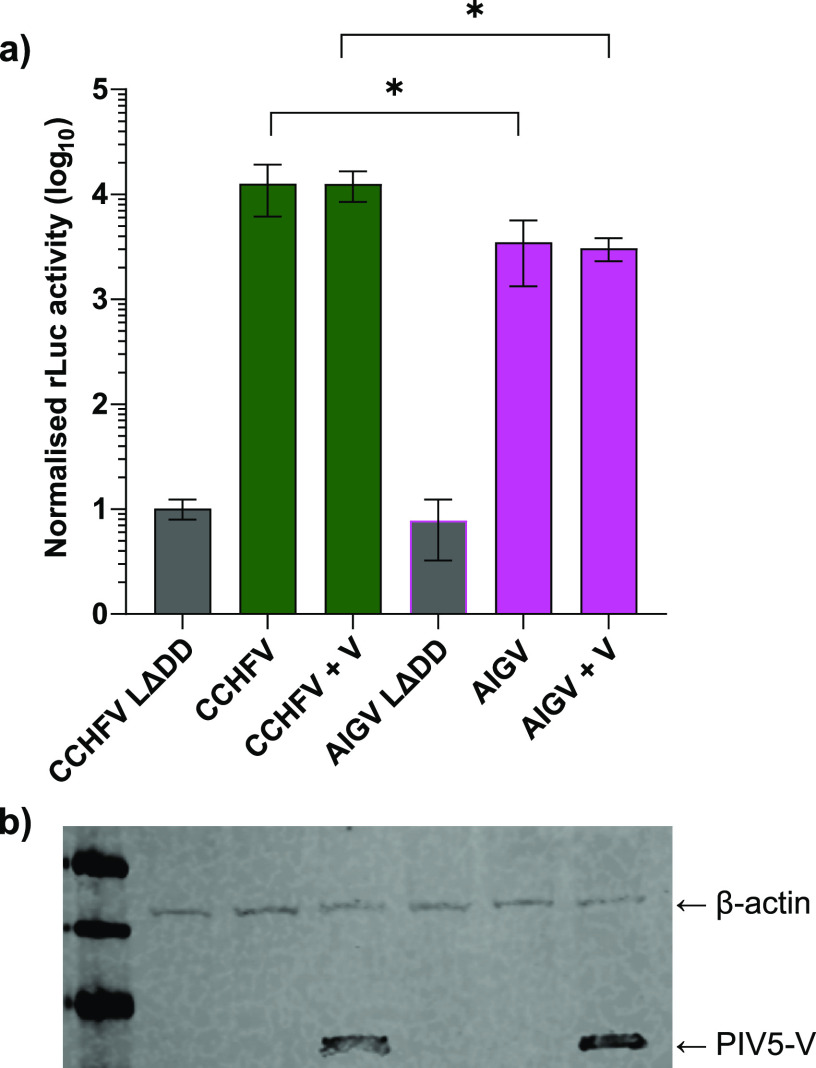
Minigenome assay in the presence of PIV5-V. (a) Huh7 cells were transfected with plasmids containing an L segment *Renilla* luciferase minigenome with a hammerhead ribozyme, N protein, L protein, wild-type T7 RNA polymerase, and a constitutively expressed firefly luciferase. Viral sequences were derived from either CCHFV (green) or AIGV (pink), and assays were carried out in the presence of a plasmid expressing PIV5-V protein (+V) or amounts normalized with an equivalent amount of empty vector. The cells were lysed 48 h posttransfection, and dual-luciferase assays were carried out with *Renilla* luciferase activity normalized to firefly luciferase activity. For the data presented, *n* = 6, with error bars showing standard deviation. Statistical analysis was carried out using an unpaired *t* test. *, *P* < 0.05. (b) Lysates from the cells in panel a were analyzed via SDS-PAGE and Western blotting and probed with anti-V5 and β-actin primary antibodies. Near-infrared fluorophore-conjugated secondary antibodies were used for detection with the Li-Cor Odyssey system. Western blot lanes correspond to the conditions in panel a.

The CCHFV L protein is known to have a number of nontraditional polymerase functions. Although not all of these functions have been characterized, the most studied region is the N-terminal OTU domain and its activity in counteracting the activity of type I interferon (IFN) (7, 9, 20). It is possible that the difference in the activity of CCHFV and AIGV L proteins is due to differences in the ability of these two proteins to modulate the IFN response. To examine if this was the case, we carried out minigenome assays with CCHFV and AIGV components in the presence or absence of a plasmids expressing the V protein of parainfluenza virus 5 (PIV5-V). As PIV5-V is able to block both IFN induction (21–23) and IFN signaling (24), any difference in IFN modulatory effect of the L proteins would be ironed out. Despite clear expression of PIV5-V (Fig. 6b), no difference in reporter gene activity was seen between CCHFV or AIGV minigenome assays with or without PIV5-V coexpression, and consequently a clear difference between the CCHFV and AIGV minigenomes was seen in both settings (Fig. 6a). Taken together, these data show that the L protein of AIGV mediates lower levels of gene expression than the CCHFV L protein, and the cause of this differential activity is distinct from the ability of the L protein to modulate the IFN response.

## DISCUSSION

Comparing AIGV with the CCHFV reference strain in our life cycle modeling systems, we have demonstrated a clear difference in the ability of the L protein to mediate viral gene expression. Although the exact mechanism of this reduced gene expression was not fully investigated, it is distinct from the IFN-antagonizing activity of the OTU domain.

While the IFN-antagonizing effect of the OTU domain has been excluded as the cause of the differential activity, this does not exclude other functions of the OTU domain. Indeed, some evidence exists to suggest this may in fact be the case. While not fully elucidated, these functions involve the deconjugation and turnover of ubiquitin and ISG15 (8, 9). The OTU domain of AIGV is less sensitive than the CCHFV domain to a modified ubiquitin variant (Ubv-CC4) (8). This variant acts as an OTU inhibitor, binding the same site in the OTU domain as wild-type ubiquitin (8). This suggests differences in the ability of the AIGV OTU domain to bind ubiquitin compared to the CCHFV domain, with potential consequences on the regulatory activity of the OTU domain (25). As well as removing ubiquitin, the OTU domain also deconjugates ISG15 from proteins, which acts as a positive regulator of CCHFV polymerase activity (9). The binding site of ISG15 in the OTU domain shares common sites with the binding ubiquitin (26, 27): consequently, it is possible that the apparent reduction in Ubv-CC4 binding is also indicative of reduced ISG15 binding and turnover.

It must also be noted that the differences between the two L proteins may be due to an uncharacterized functional domain of the L protein. Relative to other L proteins of the *Bunyavirales* order, the orthonairovirus L proteins are expanded in length throughout the protein, with the additional amino acids between conserved regions showing no clear homology to other known proteins. The function of these regions has not been explored yet, and differences within these regions may contribute to the difference in gene expression we observed.

Although not examined here, our data can be interpreted as being consistent with AIGV being a less virulent virus. Indeed, there is precedent for similar results derived from life cycle modeling systems correlating well with viral pathogenicity with hemorrhagic fever-causing viruses. A study examining the molecular basis for Reston virus (RESTV) attenuation relative to Ebola virus (EBOV) identified slower viral RNA synthesis mediated by the viral ribonucleoprotein complex to be a potential cause of attenuation (28). These data comparing RESTV and EBOV in life cycle modeling systems correlated well with *in vitro* growth and observed pathogenicity in animal models (28). Although life cycle modeling data correlated well with other hemorrhagic fever-causing viruses, both *in vitro* and *in vivo* data are lacking for AIGV. Both sets of data will be important as the correlates of CCHFV/AIGV virulence, and the determinants of these are far from clear. Although high viral load is correlated with poor outcomes during CCHFV infections, the relationship between viral growth fitness in cell culture and viral fitness in natural infections is not always simple (29). Indeed, for Nairobi sheep disease virus, a related member of the *Orthonairovirus* genus, an inverse relationship between cell culture propagation and virulence in sheep has been demonstrated (30).

It is also possible that any potential difference in pathogenicity is multifaceted. A recent study published by Hue et al. examined the Malko Tarnovo strain (MT-1303) of AIGV (previously classified as CCHFV genotype VI/Europe II) (31). The MT-1303 strain is another AIGV recovered from sequences acquired directly from an infected tick. As well as demonstrating the requirement of a single amino acid in GPC to allow infection of human cells, the study also demonstrated that the MT-1303 L protein produced lower levels of reporter gene activity than the CCHFV IbAr10200 strain (31). Our data are consistent with this study and suggest that lower L protein activity is a common feature of AIGV and show that even once the cell entry species barrier is overcome, AIGV may still be less virulent.

It is also interesting to note that while the AIGV AP92 strain GPC used in this study contains the G1116R change that Hue et al. showed to be required to mediate efficient entry into human cells, a number of amino acid differences exist, which likely explain the enhancing effect of the AP92 GPC relative to CCHFV, which was not seen with the MT-1303 GPC. Further investigation of these amino acids within GPC may shed light on both the molecular biology of CCHFV GPC and also the mechanisms of tissue culture adaption to better inform CCHFV studies.

Determination of the mechanistic causes of the altered gene expression profile of the AIGV L remains to be carried out, as does determination of the effect of these differences on viral fitness. It will also be important to empirically determine if the AIGV AP92 strain and other isolated viruses are less virulent strains in relevant *in vivo* studies and, if this is the case, the exact determinants of this. Improved knowledge of the AIGV would help to assess the risks posed from AIGV emergence and its interaction with CCFHV, helping to focus surveillance studies. Improved knowledge of the determinants of pathogenicity will help us to understand the molecular biology of both CCHFV and AIGV and will hopefully inform countermeasures in the shape of antiviral drugs as well as vaccine development.

## MATERIALS AND METHODS

### Cells.

Huh7 human hepatoma cells (kindly provided by Stephan Becker, Philipps-University Marburg) were maintained in Dulbecco's modified Eagle medium (Lonza) supplemented with 10% fetal bovine serum, 1% l-glutamine, and 1% penicillin-streptomycin antibiotic mixture (Thermo Fisher). Cells were grown in a humidified incubator at 37°C in 5% CO_2_.

### Plasmids.

The original CCHFV IbAr10200 L segment-based *Renilla* luciferase minigenome-containing plasmid (pT7riboSM2_vL_Ren) has been previously described, as have constructs encoding IbAr10200 N protein (pCAGGS_N, referred to here as pCAGGS-10200-N), IbAr10200 GPC (pCAGGS_GP, referred to here as pCAGGS-10200-M), and IbAr10200 L protein (pCAGGS_V5_L_wt), as well as the polymerase-deficient variant control (pCAGGS_V5_LΔDD) (5, 32). The T7 RNA polymerase expression plasmid (pCAGGS_T7) has also been previously reported, while the plasmid expressing the codon-optimized T7 RNA polymerase (pCAGGS-T7opt) was a gift from Benhur Lee (Icahn School of Medicine at Mount Sinai) (Addgene plasmid 65974). The plasmid constitutively expressing firefly luciferase (pGL3-luc) was purchased from Promega. The plasmid expressing the V protein of parainfluenza virus 5 (PIV5-V) (pCAGGS_PIV5-V) was a gift from Steve Goodbourn (St. George’s, University of London).

To produce the hammerhead ribozyme-incorporating IbAr10200 minigenome, a DNA fragment incorporating the optimal T7 RNA polymerase promoter and a hammerhead ribozyme followed by the IbAr10200 L segment 5′ NCR located between PciI and BglII restriction sites was synthesized (Eurofins Genomics). Using the PciI and BglII sites, the fragment was introduced to pT7riboSM2_vL_Ren using traditional molecular biology techniques to produce pT7ribo_10200_L_HHR_rLuc.

To produce the plasmid incorporating the AIGV (AP92 isolate) L NCRs, two DNA fragments were synthesized (Eurofins Genomics): one encoded the optimal T7 RNA polymerase promoter and a hammerhead ribozyme followed by the AP92 L segment 5′ NCR located between PciI and BglII restriction sites, while the other encoded the 3′ NCR from the AP92 L segment followed by a hepatitis D virus 1 ribozyme and T7 RNA polymerase terminator between KpnI and XbaI restriction sites. These two fragments were incorporated into pT7riboSM2_vL_Ren, replacing the IbAr10200 sequence, resulting in the plasmid pT7ribo_AP92_L_HHR_rLuc.

To clone the AP92 L open reading frame (ORF), reverse transcription-PCRs (RT-PCRs) (RT with RevertAid reverse transcriptase from Thermo Fisher and PCR with Q5 high-fidelity DNA polymerase from New England Biolabs) were carried out with RNA extracted from AP92-infected cells to produce overlapping DNA fragments covering the entire ORF. The primers used incorporated the endogenously encoded NdeI, AvrII, BamHI, ApaI, and BssSI restriction sites. The 5′ and 3′ termini of the ORF were amplified using primers incorporating an Esp3I and XhoI restriction site, respectively, to allow final incorporation into the expression vector. The RT-PCRs resulted in six fragments, which were blunt end cloned into pJET1.2 (Thermo Scientific). Sanger sequencing confirmed the correct DNA sequence with reference to the GenBank sequence (accession no. DQ211612.1). Using traditional molecular biological techniques, the resulting six plasmids were used to construct two plasmids: one (pJ-AP92-L-low) contained the N-terminal 5,429 bp of the AP92 L ORF located between the Esp3I and BamHI restriction sites, while the other (pJ-AP92-L-top) contained the C-terminal 6,434 bp between the BamHI and XhoI restriction sites. The plasmids were digested with either Esp3I and BamHI or BamHI and XhoI, giving rise to two fragments that were incorporated into pCAGGS_N-V5_GG-Esp3I, a pCAGGS plasmid encoding an N-terminal V5 tag. The resulting plasmid, known as pCAGGS_N-V5-AP92-L, encoded the L protein of AP92 with an N-terminal V5 tag.

A polymerase-inactive control plasmid was produced by the deletion of six bp in pJ-AP92-L-top, which results in a two amino-acid deletion at positions 2571 and 2518. The resulting plasmid was used in combination with pJ-AP92-L-low as described above to produce pCAGGS_N-V5-AP92-LΔDD.

To produce the AP92-M expression plasmid, RT-PCRs (RT with RevertAid reverse transcriptase from Thermo Fisher and PCR with Q5 high-fidelity DNA polymerase from New England Biolabs) were carried out on RNA extracted from AP92-infected cells using overlapping primers incorporating the endogenously encoded XbaI and AvrII restriction sites as well as primers complementary to the 5′ and 3′ termini of the ORF, which included overhanging ClaI and XhoI restriction sites, respectively. After the RT-PCR, the expected 1,943-, 2,389-, and 825-bp fragments were isolated and combined in a second round of PCR (with Q5 high-fidelity DNA polymerase from New England Biolabs) using the outer ClaI- and XhoI-incorporating primers, which gave rise to the expected 5,110-bp DNA fragment. Using the ClaI and XhoI sites, the complete AP92 M-encoding DNA fragment was incorporated into pCAGGS and was subsequently Sanger sequenced to confirm the correct sequence with reference to the GenBank sequence (accession no. DQ211625.1).

AP92 N was cloned using primers incorporating a 5′ ClaI restriction site and 3′ XhoI for RT-PCR (RT with RevertAid reverse transcriptase from Thermo Fisher and PCR with Q5 high-fidelity DNA polymerase from New England Biolabs) to amplify the AP92-N ORF. Using the ClaI and XhoI sites, the ORF was incorporated into pCAGGS to produce pCAGGS-AP92-N. The final plasmid was Sanger sequenced (Eurofins Genomics) and confirmed to incorporate the correct sequence with reference to the GenBank sequence (accession no. DQ211638.1).

### tcVLP assays.

Huh7 cells were seeded into 6-well plates at 2 × 10^5^ cells per well 18 h prior to transfection. Each well was transfected with 600 ng of an L-expressing plasmid (pCAGGS_V5_L, pCAGGS_N-V5-AP92-L, or the respective negative-control LΔDD plasmid), 200 ng of N-expressing plasmid (pCAGGS-10200-N or pCAGGS-AP92-N), 500 ng of GPC-expressing plasmid (pCAGGS-10200-M or pCAGGS-AP92-M), 200 ng of a minigenome-containing plasmid (pT7riboSM2_vL_Ren, pT7ribo-10200_L_HHR-rLuc, or pT7ribo-AP92_L_HHR-rLuc), and 500 ng of a T7 RNA polymerase-expressing plasmid (pCAGGS_T7 or pCAGGS_T7opt) alongside 200 ng of pGL3-Luc as a transfection control using the GeneJammer transfection reagent (Agilent). When plasmid amounts varied, empty pCAGGS was included to equalize the amounts of transfected DNA. Four hours posttransfection, the media were exchanged for fresh complete media. Supernatants containing tcVLP were collected 72 h posttransfection and centrifuged, while cell lysates were collected in passive lysis buffer (Promega). *Renilla* and firefly luciferase activities in the cell lysates were measured using a dual-luciferase reporter assay system (Promega), with values measured on a Tecan Infinite M200 Pro plate reader.

Treatment of cells with tcVLP-containing supernatants was carried out under one of two conditions. Under the first condition, as “indicator cells,” Huh7 cells seeded at 2 × 10^5^ cells per well in a 6-well plate 18 h previously were transfected with 600 ng of pCAGGS_V5_L, 200 ng of pCAGGS-10200-N, and 200 ng of pGL3-luc as described above. After 24 h, media were removed, washed with phosphate-buffered saline (PBS), and treated with tcVLP-containing supernatant. After a 1-h incubation at 37°C with periodic rocking, fresh media were added on top of the inoculum. The cells were lysed 24 h post-tcVLP treatment, and luciferase assays were carried out as described above.

Alternatively, an equivalent plate of Huh7 cells was transfected as described above with 500 ng of GPC-expressing plasmid (pCAGGS-10200-M or pCAGGS-AP92-M) included in the transfection mix. Treatment with tcVLPs was carried out as described above for indicator cells. Supernatants were collected 72 h post-tcVLP treatment and clarified by centrifugation. Luciferase assays were carried out on cell lysates as described above.

### Minigenome assay.

Minigenome assays were carried out by transfecting Huh7 cells, seeded 18 h previously at 2 × 10^5^ cells per well in a 6-well plate, with 600 ng of an L-expressing plasmid (pCAGGS_V5_L, pCAGGS_N-V5-AP92-L, or the respective negative-control LΔDD plasmid), 200 ng of N-expressing plasmid (pCAGGS-10200-N or pCAGGS-AP92-N), 200 ng of a minigenome-containing plasmid (pT7riboSM2_vL_Ren, pT7ribo-10200_L_HHR-rLuc, or pT7ribo-AP92_L_HHR-rLuc), and 500 ng of a T7 RNA polymerase-expressing plasmid (pCAGGS_T7 or pCAGGS_T7opt) alongside pGL3-Luc as a transfection control. Transfections were carried out using the GeneJammer transfection reagent (Aligent) with an exchange of media 4 h posttransfection. After 48 h, cell lysates were collected in passive lysis buffer (Promega), and the *Renilla* and firefly luciferase activities were measured using the dual-luciferase reporter assay system (Promega), with values measured using a Tecan Infinite M200 Pro plate reader. *Renilla* luciferase values were normalized to firefly luciferase activity.

### Western blot.

Lysates from luciferase assays were mixed with 4× SDS sample buffer (10 μg/mL bromophenol blue, 40% glycerol, 20% β-mercaptoethanol, and 125 mM SDS) and heated at 95°C for 10 min. The samples were analyzed on a 12.5% SDS-PAGE gel with subsequent semidry Western blotting using an ethanol-containing blotting buffer [48 mM Tris(hydroxymethyl)aminomethane, 39 mM glycine, 20% [vol/vol] ethanol in H_2_O] and a nitrocellulose membrane (Amersham). Blots were blocked with 7% (mass/vol) skim milk powder in PBS. Blots were stained using mouse anti-V5 (Thermo Fisher) and rabbit anti-β-actin (Sigma-Aldrich) primary antibodies with goat anti-mouse IgG conjugated with IRDye 800CW (Li-Cor) and goat anti-rabbit IgG conjugated with IRDye 680RD (Li-Cor) used as secondary antibodies. Blots were imaged using Li-Cor Odyssey CLx system.
